# *In vitro* and *in vivo* evaluation of photo-induced antileishmanial activity of indocyanine green-loaded nanomicelles

**DOI:** 10.22038/ijbms.2025.82333.17807

**Published:** 2025

**Authors:** Shirin Jalili, Jafar Mosafer, Seyed Amin Mousavi Nezhad, Ameneh Sazgarnia, Mohammad Ali Mohaghegh, Mehdi Hoseini

**Affiliations:** 1 Institute of Police Equipment and Technologies, Policing Sciences and Social Studies Research Institute, Tehran, Iran; 2 Research Center of Advanced Technologies in Medicine, Torbat Heydariyeh University of Medical Sciences, Torbat Heydariyeh, Iran; 3 Health Sciences Research Center, Torbat Heydariyeh University of Medical Sciences, Torbat Heydariyeh, Iran; 4 Research Center for Life & Health Sciences & Biotechnology of the Police, Directorate of Health, Rescue & Treatment, Police Headquarters,Tehran, Iran; 5 Medical Physics Research Center, Mashhad University of Medical Sciences, Mashhad, Iran; 6 Department of Laboratory Sciences, School of Paramedical Sciences, Torbat Heydariyeh University of Medical Sciences, Torbat Heydariyeh, Iran; 7 Department of Radiology Technology, School of Paramedical Sciences, Torbat Heydarieh University of Medical Sciences, Torbat Heydarieh, Iran; # These authors contributed eqully to this work

**Keywords:** Leishmania major, Nanomedicine, Photodynamic therapy (PDT), Photosensitizer, Photothermal therapy (PTT)

## Abstract

**Objective(s):**

Due to its low toxicity and high absorbance in the range of 650 to 900 nm, indocyanine green (ICG) has garnered significant attention for its applications in photodynamic therapy (PDT) and photothermal therapy (PTT). However, its tendency to aggregate in aqueous environments limits its efficacy in both* in vitro* and* in vivo* applications. Encapsulating ICG in a biocompatible nanomicelle can improve its aqueous stability and photophysical properties. The present study investigated the synergistic effect of ICG-loaded nanomicelles upon irradiation by an 808-nm laser on *Leishmania major* (*L. major*) parasites.

**Materials and Methods:**

Initially, a nanomicelle comprised ICG was synthesized and characterized. Then, the temperature increase during irradiation and promastigote viability were evaluated *in vitro*. Subsequently, the prepared samples’ *in vitro* dark toxicity and phototoxicity were assessed via the MTS assay. Finally, the *in vivo *antileishmanial efficacy of the ICG-loaded nanomicelles formulation was investigated in BALB/c mice.

**Results:**

The absorbance of ICG-loaded nanomicelles at 808 nm was more than 2 times greater than Free-ICG. Also, the prepared formulation exhibited a mean diameter of ~25 nm and a zeta potential of -2.3 ± 1 mV. The combination of ICG-loaded nanomicelles and 808 nm laser with a power density of 2.5 W cm^−2^ led to a significant reduction in the survival rate of promastigotes and lesion size of infected mice compared to control groups.

**Conclusion:**

The PDT/PTT mediated by ICG-loaded nanomicelles can be considered a promising and efficient therapeutic method for *L. major*, as it is inexpensive, safe, and easy to implement.

## Introduction

Leishmaniasis is a neglected infectious disease caused by different species of flagellated protozoan *Leishmania* and is transmitted by the bite of female *phlebotomine* sandflies (1, 2). Leishmaniasis is one of the top 10 neglected tropical diseases globally, with over 12 million people affected. It is endemic in 99 countries, with cutaneous leishmaniasis (CL) present in 89 countries and visceral leishmaniasis (VL) in 80 countries. In 71 countries, both CL and VL are endemic. This leads to more than 20,000 to 30,000 deaths annually in the 99 endemic countries (3). Although kala-azar, or VL, is the most lethal type and causes death if left untreated, CL is the most common type of leishmaniasis (4). This disease significantly burdens human health and is a major global problem. CL is commonly attributed to three species of *Leishmania*: *Leishmania major* (*L. major*), *L. tropica*, and *L. infantum*. Specifically, *L. major*, a zoonotic species, is responsible for causing CL characterized by suppurating wounds. This condition often presents challenges in treatment due to its frequent association with other microbial infections (5, 6).

Common antiparasitic drugs such as pentavalent antimony, miltefosine, isothionic pentamidine, and amphotericin B are used to treat leishmaniasis. Some of these drugs are relatively expensive and could cause severe side effects and parasite resistance, and they are not always recommended for treatment. Therefore, developing new drugs or alternative treatments is necessary for leishmaniasis (7-10).

In recent years, physical methods such as cryotherapy, hyperthermia, electrolysis, and photodynamic therapy (PDT) have been suggested to treat leishmaniasis (11-14). Photothermal therapy (PTT) uses near-infrared (NIR) light to induce hyperthermia through the absorption of light by a photothermal agent (15). PDT is a safe, low-cost, and minimally invasive treatment modality with minor side effects for several diseases. It involves a combination of non-toxic dyes called photosensitizers (PS) and the administration of a specific wavelength of light to induce cellular effects by generating reactive oxygen species (ROS). PDT can be an alternative treatment for local infections like CL, as it does not induce resistance due to the interaction of ROS with several cellular structures through multiple mechanisms (16-21).

Indocyanine green (ICG) has been approved by the US Food and Drug Administration (FDA) for medical applications. ICG is a hydrophilic, negatively charged tricarbocyanine dye with almost no toxicity. Its high absorption in the NIR region makes it a reliable PS for combined PDT/PTT. However, its poor aqueous stability and lack of targeting limit its application (22-24).

Due to their small size and high surface-to-volume ratio, nanoparticulated drug delivery systems (NDDSs), such as nanomicelles, can improve the therapeutic efficacy of drugs. Additionally, nanoencapsulation often allows for lower drug dosages, thereby minimizing potential side effects (25-27). As delivery agents for PSs, nanomicelles offer several advantages over conventional formulations, including improved solubility, reduced toxicity, improved pharmaceutical activity, and enhanced stability and protection against physical and chemical degradation (28, 29).

To our knowledge, no study has been conducted on applying ICG-loaded nanomicelles to treat cutaneous leishmaniasis. Therefore, this study aimed to introduce a nanostructure that improves the photophysical properties of ICG and investigate the synergistic effect of PDT and PTT on leishmaniasis in the presence of ICG-loaded nanomicelles.

## Materials and Methods

### Chemicals

ICG was purchased from ACROS (USA, 412541000) and MTS/PMS (3-(4,5 dimethylthiazol-2-yl)-5-(3-carboxymethoxyphenyl)-2-(4-sulfophenyl)-2H-tetrazolium/phenazine methosulfate), penicillin, trypsin–EDTA, and streptomycin were purchased from Sigma‒Aldrich (St. Louis, MO, USA). Roswell Park Memorial Institute-1640 (RPMI-1640) medium and fetal bovine serum (FBS) were obtained from Gibco (Invitrogen Corporation, Carlsbad, CA, USA). Surfactants (TWEEN-80 and SPAN-80) were procured from Sigma‒Aldrich (St. Louis, MO, USA). All other chemicals were of analytical grade.

### Preparation and characterization of ICG-loaded nanomicelles

ICG-loaded nanomicelles prepared through a simple equilibrium method in which ICG and two nonionic surfactants with two completely different hydrophilic–hydrophobic balances (HLBs) were added to the aqueous medium during stirring. In this way, a solution of 2 μM ICG in micelles was prepared with SPAN-80 (HLB of 4.3) with 60% and TWEEN-80 (HLB of 15) with 40% of the total portion so that an HLB of 8.58 was obtained. The final concentrations of SPAN-80 and TWEEN-80 in the formulation were 2.5 mM and 1.5 mM, respectively.

To investigate the role of the prepared nanomicelles in improving the optical properties of ICG, UV–vis absorption spectra (UNICO 2100-UV, China) with 5 nm accuracy were recorded (the concentration of ICG in both samples was 2 μM). The size distribution and zeta potential of the ICG-loaded micelles were measured by a SZ-100 instrument (HORIBA, Japan) at 25 °C equipped with a helium-neon (He-Ne) laser (633 nm). To determine the emission spectrum of the 808 nm laser, a spectrometer (AvaSpec, Netherlands) was used.

### Aqueous photophysical stability study

The influence of aqueous media on the deactivation of ICG in the dark was assessed for Free-ICG and ICG-loaded nanomicelles at 25 °C. The ICG concentration in each sample was 2 μM. Each sample was placed in a glass vial with aluminum foil to prevent light exposure. The absorption spectrum of the samples was monitored daily for five days.

### In vitro studies

*Parasite cultivation*

The virulence of *L. major* (MRHO/IR/75/ER) was maintained by a continuous passage in BALB/c female mice. The spleen biopsy of infected mice was transferred to a biphasic Novy–MacNeal–Nicolle (NNN) medium to isolate amastigotes. After transforming amastigotes to promastigotes, they were cultured in RPMI-1640 supplemented with 15% FBS and 1% pen-strep at 28 °C incubator for mass cultivation (12, 30, 31).

*In vitro cytotoxicity measurements*

The dark toxicity of Free and ICG-loaded nanomicelles on *L. major* parasites was investigated at five concentrations. At first, the promastigotes were seeded in triplicate in 96-well plates at a density of 10^6^ parasites/ml in 100 μl and incubated at 28 °C for 24 hr. Then, the plate was incubated with different concentrations of ICG (10, 20, 40, 80, and 100 μM) at 28 °C for another 24 hr. Next, the samples were centrifuged at 3200 × g for 10 min at 4 °C, and the supernatant was removed. After that, RPMI-1640 culture medium (without phenol red) supplemented with 10% FBS was added to each well and incubated for 48 hr. To determine the cell survival rate, a mixture of the resulting MTS/PMS solution (at a ratio of 5:1) with a volume of 20 μl was added to each well (32). Finally, after 3 hr, light absorption at a wavelength of 492 nm was measured using a Stat Fax 2100 microplate reader (Awareness Technology, Palm City, FL, USA) equipped with a printer (LQ-300 EPSON, Nagano, Japan). The following formulation was used to determine the cell survival rate: 

$\mathrm{Cell survival rate}=\frac{\left(\mathrm{Optical density of treated sample}-\mathrm{optical density of blank}\right)}{\left(\mathrm{Optical density of untreated control}-\mathrm{optical density of blank}\right)}\times 100$

*Temperature measurement under NIR laser irradiation*

Considering the results obtained in the viability assay, the concentration of 100 µM was selected to evaluate the photothermal response of ICG. One milliliter of each of the three samples, namely, pure water, Free-ICG, and ICG-loaded nanomicelles, in conical tubes was irradiated with an 808 nm continuous-wave NIR diode laser (MDL-III-808, CNI Laser, China) at a radiant energy fluence rate (power density) of 2.5 W cm^−2^ for 10 min (fluence of 1500 J cm^-2^). The temperature of solutions was monitored after NIR irradiation by an infrared thermal imaging camera with an accuracy of 0.05 °C (Testo 882, Germany).

### In vitro ICG-NIR irradiation

To determine the cytotoxicity of ICG with/without NIR irradiation, promastigotes were incubated for 24 hr in darkness with 100 μM of ICG (Free-ICG and ICG-loaded nanomicelles) at 28 °C. After washout, the parasites were transferred to a 96-well plate and irradiated from the bottom upward using the NIR laser at a power density of 2.5 W cm^−2^. The exposure times for all groups were 0, 5, and 10 min, corresponding to fluences of 0, 750, and 1500 J cm^-2^, respectively. Following irradiation, 100 µl of fresh culture medium was added. After 24 hr, promastigotes viability was measured by the MTS assay described above. Each experiment included three controls: (1) no treatment, (2) treatment with 100 μM ICG in the dark, and (3) treatment with NIR irradiation only.

### In vivo studies

*Animals*

Female BALB/c mice were purchased from the Pasteur Institute, Karaj Branch, Iran, and used at 6–8 weeks of age (n=33, mean weight 20 g, standard deviation 5 g). The animals were maintained under specific pathogen-free conditions at 22–25 °C with a 12 hr light/dark cycle. All animal procedures complied with Animal Care and Use Committee-approved protocols of Shahid Beheshti University of Medical Sciences.

*Induction of leishmaniasis and ICG-NIR irradiation in BALB/c mice*

The 3rd passage of promastigotes in the fixed growth phase was used to infect the mice. After proliferation and harvesting, the parasites were washed with PBS, and 100 μl of solution containing live promastigotes (4 × 10^7^/ml) was subcutaneously injected into the right hind paw of each animal. After approximately 20 days, the wound appeared, and the animals were randomly separated into six groups (4 per group). The groups of animal were defined as follows: 1) control (untreated mice), 2) ICG-Free, 3) ICG-loaded nanomicelles, 4) only laser irradiation, 5) ICG-Free and laser irradiation, and 6) ICG-loaded nanomicelles and laser irradiation. In groups 2, 3, 5, and 6, a suspension of ICG (with/without nanomicelles) in PBS (100 μg ml^−1^, 100 μl, ∼0.5 mg kg^−1^ mouse^−1^) was locally injected in lesions of the animals, while 100 μl PBS was injected to the lesion of the mice in groups 1 and 4. Immediately after injection, the lesion of the mice in groups 4, 5, and 6 was exposed to laser irradiation at a power intensity of 2.5 W cm^-2^ for 10 min (fluence of 1500 J cm^-2^). After irradiation, the mice were returned to the colony**.** For *in vivo* therapeutic efficacy evaluation of the different formulations, the changes in the relative diameter of the induced lesion between the groups were recorded every 3 days by a digital caliper, and their changes were normalized compared to the day before the treatment for a total of 30 days following the treatment.

### Statistical analysis

The data were derived from at least three independent experiments and are presented as the mean ± standard error of the mean. Data from different stages of the study were entered into SPSS version 26 software (IBM Corp., Armonk, NY, USA) for statistical analysis. *P*-values less than 0.05 were considered statistically significant.

Moreover, using a linear equation to fit the toxicity graph of ICG with and without nanomicelles, the IC_20_ (a concentration of ICG that inhibits 20% of promastigotes) was calculated.

To compare the efficiency of the *in vitro* treatment, the combination index (CI) was defined to compare the groups. For this purpose, possible synergistic effects in treatment were calculated in the presence of experimental groups and laser irradiation. In this way, if two factors, A and B, affect parasite survival separately or in combination with each other, the CI is defined as follows:

$\mathrm{CI}=\frac{V_{A}\times V_{B}}{V_{\mathrm{AB}}}$

Where V_A_, V_B_, and V_AB_ are the parasite viabilities under the effects of A, B (alone), and their combination, respectively. Here, CI > 1, CI = 1, or CI < 1 indicate the dominance of synergistic, additive, or antagonistic effects of agents A and B, respectively (33).

Additionally, to compare two treatment groups, one with nanomicelles and the other without nanomicelles (both groups were irradiated), the relative lethal synergism (RLS) index was used, which is equal to the ratio of cell death following treatment in the presence of nanomicelles to cell death after using the same treatment method but in the absence of nanomicelles. In this case, if the RLS is greater than 1, there is a synergistic effect (34).

## Results

### Characterization of ICG-loaded nanomicelles

**Figure 1:**
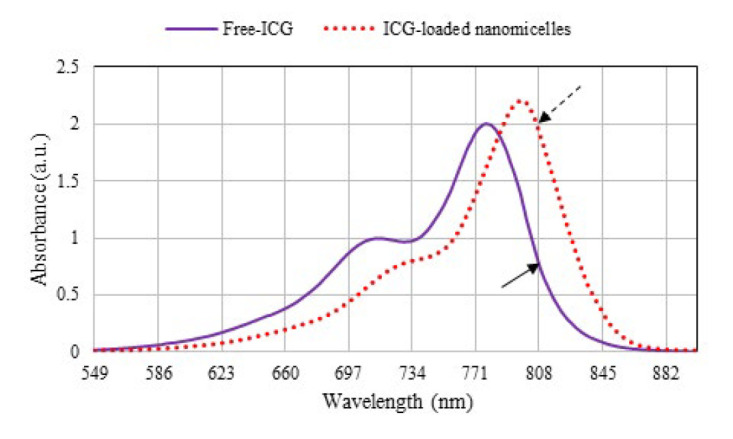
UV‒vis spectra of Free-ICG and ICG-loaded nanomicelles

The results in Figure 1 indicated that the absorbance of Free-ICG attains a peak at 774 ± 1 nm such that it shifted to 802 ± 1 nm when loaded into nanomicelles. As shown in Figure 1, when ICG was formulated into the nanomicelles, its absorption at 808 nm had markedly increased compared with that of Free-ICG.

**Figure 2:**
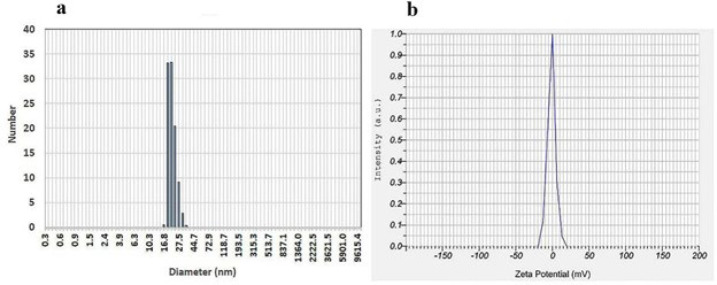
Particle size (a) and zeta potential (b) of ICG-loaded nanomicelles

According to the results obtained from the particle size analyzer instrument, the nanomicelles had a narrow size of approximately 25 ± 10 nm (Figure 2 a) and a polydispersity index (PDI) of 0.65. As shown in Figure 2 b, the nanomicells had a zeta potential of -2.3 ± 1 mV.

### Aqueous photophysical stability study

**Figure 3:**
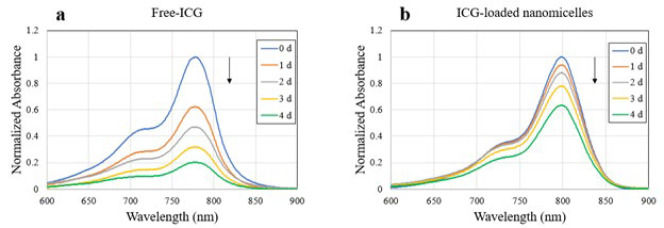
Normalized absorption spectrum of (a) Free-ICG and (b) ICG-loaded nanomicelles measured for 5 days

Data indicated that the absorbance of Free-ICG steadily decreased over time, with 80% of the absorption peak lost after 5 days of storage (Figure 3a). However, the absorbance of ICG-loaded nanomicelles decreased minimally, and 65% of the original absorbance was still present on day 5 (Figure 3b).

### In vitro cytotoxicity measurements

**Figure 4:**
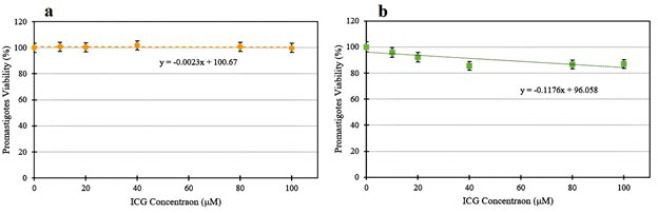
Percentage of *Leishmania major* promastigotes viability in different concentrations of (a) Free-ICG and (b) ICG-loaded nanomicelles

As shown in Figure 4 a, the difference in the average percentage of cell survival in the groups containing Free-ICG was insignificant compared with that in the control group (*P*>0.05). According to Figure 4 b, the viability of parasites in ICG-loaded nanomicelles at concentration of 10 μM did not induce any significant decrease compared to the control group (*P*=0.152). However, at concentrations higher than 10 μM, viability decreased significantly in *L. major* promastigotes compared to the control group (*P*<0.02). Based on the above results, the IC_20_ concentration in Free-ICG groups was 390 μM, and in ICG-loaded nanomicelles groups, 102 μM. Therefore, the optimal concentration of ICG was considered to be 100 μM.

### Temperature measurements under NIR laser irradiation

**Figure 5:**
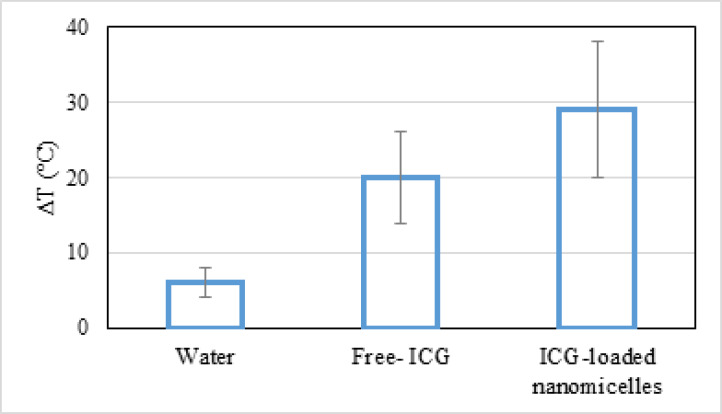
Temperature increase (ΔT) of water, Free-ICG, ICG-loaded nanomicelles (ICG concentration= 100 μM) under irradiation of 808 nm laser (10 min, 1 W) with respect to the control (no irradiation) as detected with a thermal camera

As shown in Figure 5, the temperature of blank water increased to 6 °C after 10 min of NIR laser irradiation (fluence of 1500 J cm^-2^), while for the Free-ICG and ICG-loaded nanomicelles, it increased considerably during laser irradiation. After 10 min of irradiation, the temperature of the Free-ICG and the ICG-loaded nanomicelles increased by 20 °C and 29 °C, respectively.

### In vitro ICG-NIR irradiation

**Figure 6:**
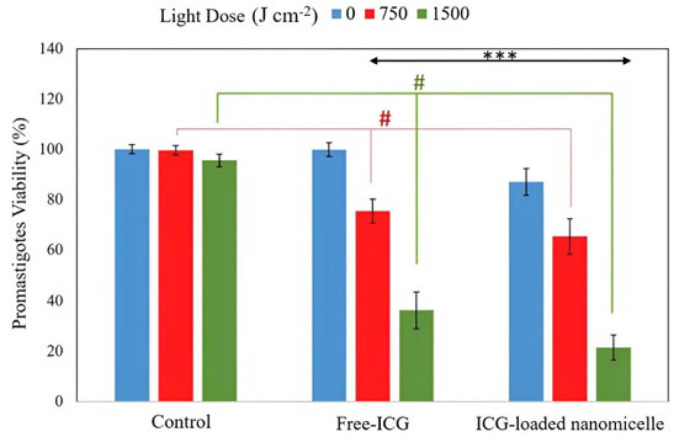
Percentage of *Leishmania major *promastigotes viability after exposure to an 808 nm laser at three different fluences and a concentration of 100 μM ICG

The results in Figure 6 showed that in the groups treated with only laser irradiation, there was no significant decrease in cell survival compared to that in the control group without laser irradiation (*P*>0.05), indicating the lack of phototoxicity of the 808 nm wavelength. Interestingly, a significant decrease in the cell survival rate was observed in Free-ICG and the ICG-loaded nanomicelles groups by increasing the fluence from 750 to 1500 J cm^-2 ^(*P*<0.001).

Additionally, the CI of the NIR laser and ICG were calculated for different fluences, as shown in Table 1.

Also, according to the results of the MTS assay, the RLS indices were calculated for the groups receiving lasers containing both Free-ICG and ICG-loaded nanomicelles at two fluences, 750 and 1500 J cm^-2^ which were 1.23 and 1.41, respectively. There was a synergistic effect since the RLS was greater than 1 for both fluences.

### In vivo antileishmanial activity

**Figure 7:**
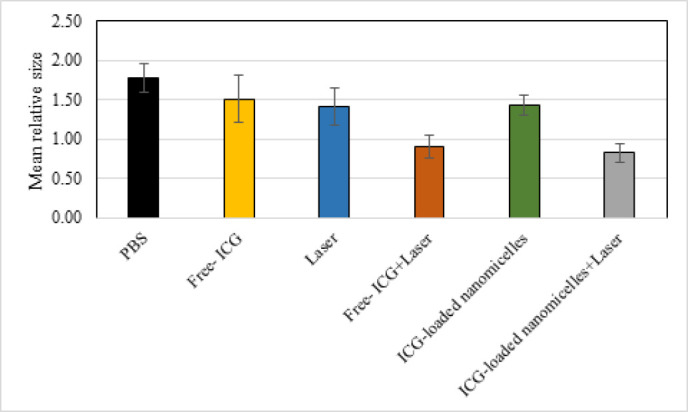
Variations in the relative surface area of the lesions in mice in different groups a) Free- ICG and b) ICG-loaded nanomicelles over consecutive days

The effects of 808 nm laser irradiation alone and in the presence of dye on right foot lesions in BALB/c mice were studied during the experimental period. The lesion sizes of the mice were monitored until the 30^th^ day to determine the rate of decrease in lesion size. At the end of the experiment, the lesion size was more than 2-fold greater in the control group than in the groups receiving ICG + laser. However, from the 9^th^ to 18^th^ days, the lesion size in the animals followed the order: ICG-loaded nanomicelles + laser < Free-ICG + laser < ICG or laser alone < control. The lesion size in the groups that received ICG-loaded nanomicelles + laser treatment noticeably decreased. In contrast, in the control group, the increase in lesion size reached more than 50% compared to that on the first day before treatment. However, during the follow-up period from the 18^th^ day until the end of the experimental period, no reduction in lesion size was observed in any of the groups; however, the lesion size was increased by approximately 40% compared to that on the first day of treatment, except in the Free-ICG and ICG-loaded nanomicelle groups that received laser irradiation (Figure 7).

**Figure 8:**
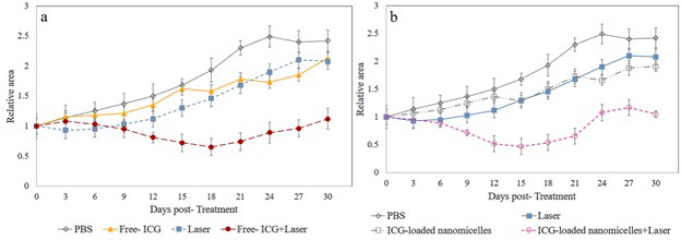
Mean relative size of lesions 30 days after treating mice with ICG and NIR laser in experimental groups

Additionally, the average relative size of the lesion was calculated within 30 days for the studied animal groups and is shown in Figure 8. Based on this analysis, the simultaneous application of ICG and the laser resulted in a significant decrease in the relative size of the lesion compared to that in the control group (*P*<0.001). Finally, although there was no significant difference in the relative size of the lesion between the two groups, namely, the Free-ICG + laser group and the ICG-loaded nanomicelles + laser group (*P*=0.45), a significant difference was observed between the groups receiving ICG and laser irradiation simultaneously and the other groups (*P*<0.008).

## Discussion

Over the past several decades, the incidence of CL has almost reached epidemic proportions. Chemotherapy is currently the primary method for controlling *Leishmania* parasites despite resistance and severe side effects on patients. In addition, both first and second-line drugs may cause bone marrow suppression, hepatotoxicity, and severe pain. Moreover, some drugs like miltefosine are expensive and not affordable in developing countries. Therefore, finding less toxic chemical and non-chemical alternatives is crucial for reducing drug complications (8, 35, 36). The PDT/PTT method has been proven to be highly effective in fighting cancer and microbial infections, as well as aiding in wound healing (37, 38). ICG, an FDA-approved photosensitizer, is a valuable PDT and PTT agent commonly used due to its hydrophilic nature, low toxicity, and high absorption in the NIR region, generating ROS and increasing local temperature (15). The use of ICG in PDT has two primary drawbacks, namely, its quick elimination from the body due to its binding to lipoproteins and its instability in aqueous solutions (39, 40). To overcome these challenges, a possible solution is to apply biocompatible and biodegradable nanoparticulate systems that can act as carriers for both lipophilic and hydrophilic drugs, such as ICG. In recent years, micellar nanostructures have emerged as potential colloidal drug carriers that can address these issues effectively. Therefore, ICG-loaded nanomicelles were synthesized using SPAN-80 and TWEEN-80 as biocompatible agents. The prepared nanoparticles were characterized and employed for further studies. According to Figure 1, the redshift of the ICG absorbance in the aqueous medium demonstrated that the aggregation of ICG decreased after it entered the nanomicelles, consistent with the data in the literature. Accordingly, the role of the prepared micellar nanostructure in improving the optical properties of ICG was confirmed (39-41). The reason for enhancement in the absorption peak of the ICG at 808 nm when loaded in nanomicelles can be attributed to the interaction of the dye with the micellar structure. The encapsulation of ICG reduces dye-dye interactions and van der Waals attraction between the dye molecules, preventing the formation of dimeric structures. Therefore, the quantum efficiency of the excited state, ultimately generating ROS and hyperthermia, can be enhanced.

The reason for choosing the SPAN-80 surfactant as the dominant surfactant in this study is its hydrophobicity, which increases the probability of passing through the phospholipid membrane of protozoa. SPAN-80 has low toxicity and a very low critical micelle concentration (CMC), which prevents premature micelle separation even in blood and tissue. The nanomicelles synthesized in this study are likely vesicles because SPAN-80 is a vesicle-forming surfactant. Additionally, TWEEN-80, as an edge-activating surfactant, is used in a suitable ratio with SPAN-80, which leads to changes in the fluidity, elasticity, and integrity of the nanomicelles (42, 43).

Additionally, as shown in Figure 2 b, the zeta potential of the nanomicelles was around zero. The nonionic nature of the used surfactants is one of the main reasons for neutralizing the zeta potential (44, 45). In a study conducted by Kirchherr *et al*., ICG was encapsulated into biocompatible surfactant micelles. Their results showed that the prepared micelles’ size, zeta potential, and PDI were 12 nm, -1.2 mV, and 0.05, respectively. Additionally, the absorption range of ICG in these micelles increased by 40% compared to that of the free ICG and showed a redshift from 779 to 798 nm (40). The differences in the absorption spectrum of micelles, their size, and their PDI can be attributed to several factors. These include the type of surfactant used, which affects its CMC, molecular structure, and HLB. In this study, the concentration of ICG was 2 μM, whereas Kirchherr *et al*. used a higher concentration of 12.8 μM. Additionally, the HLB value of the surfactant in their study ranged from 14 to 16; in our study, it was approximately 8.6.

Stability comparison of Free-ICG and ICG-loaded nanomicelles during 5 days indicates that Free-ICG completely aggregated in an aqueous solution while after it was entrapped in nanomicelles, its aggregation was significantly decreased and efficiently stabilized at room temperature (Figure 3). Similar findings have been reported elsewhere (22, 39-41, 46, 47).

An essential factor for photosensitizers in practical applications is dark toxicity. According to the results presented in Figure 4 b, the average cell survival rate at all concentrations of ICG in the group that received ICG-loaded nanomicelles (except 10 μM) was significantly lower than the control group (*P*<0.02). However, the difference in the average cell survival rate between all the other groups containing Free-ICG and the control group was insignificant (*P*>0.05), indicating that the ICG is non-toxic in physiological fluids. The effect of ICG concentrations on *Leishmania* promastigotes has not been studied so far. However, its very low dark toxicity has been reported in studies conducted on cancer cells, including U-87 MG cells (48), MCF-7 and HT-29 cells (49), KB cells (50), MC-38, and CT26 cells (51). Additionally, the micellar structure containing ICG exhibited greater toxicity than Free-ICG, suggesting that the nanomicelles are more effective at targeting *Leishmania *parasite promastigotes.

This is likely due to the hydrophobic property of the prepared nanomicelles, which increases the rate of passage through the phospholipid membrane. Accordingly, a concentration of 100 µM, which induced less than 20% inhibition of parasite growth, was used to investigate the photodynamic/photothermal effect. Interestingly, one of the main advantages of ICG over porphyrins is its minimal absorption in the 400–600 nm range, which results in lower phototoxicity to the skin (52).

The photothermal ability of ICG under 808 nm laser irradiation was investigated by monitoring the solution temperature with a thermal camera. During 10 min irradiation by a 2.5 W cm^−2^ NIR laser, the temperatures of Free-ICG and ICG-loaded nanomicelles were considerably increased. In contrast, only a slight increase in the temperature of blank water was attained (Figure 5). Also, at the same irradiation time, the temperature of ICG-loaded nanomicelles was increased compared with that of the Free-ICG sample. This could be due to the location of ICG molecules near the surfaces of nanomicelles, which reduces their aggregation in aqueous media. This structure causes an increase in absorbance and a significant redshift in the absorption spectrum of ICG, from 774 to 802 nm, to correspond with the wavelength of the NIR laser at 808 nm, thereby enhancing its photothermal conversion capability (47).

According to the *in vitro* ICG-NIR irradiation of *L. major* promastigotes, the average CI for the micellar groups was significantly greater than that of the Free-ICG group (Table 1). This indicates that the micellar nanostructure is more efficient for treatment. Additionally, both the CI and RLS calculations showed a synergistic effect in the groups containing ICG. The nanostructure’s better performance than that of Free-ICG is due to the enhancement of the absorption peak of ICG at a wavelength of 808 nm, which is 2.5 times greater for the nanomicelles than for Free-ICG. The nanomicelles were used in this study for several reasons: 1) to increase the stability of ICG and its dispersion in water, 2) to improve the photophysical and photochemical properties of the dye, and 3) to enhance uptake from the cellular membrane.

Notably, the cell survival rate decreased slightly after laser exposure in the group without medicinal agents. It has been suggested that cytochrome c oxidase (Cox) is the primary light receptor in mammalian cells. Cox has the most absorption in the visible range and some in the NIR range. This result can also be applied to the mitochondria of parasites (53). Therefore, the phototoxicity of promastigotes at an 808 nm wavelength in the absence of ICG can be rationalized.

*In vivo* studies showed that the growth of CL lesions was completely inhibited only after ICG-NIR irradiation (with and without nanomicelles) and not after NIR irradiation alone. These findings indicate that ICG is necessary as both a photothermal agent and photosensitizer for effective ICG-NIR therapy. Also, due to an increase in lesion size observed in the treatment groups from the 15^th^ day, two suggestions can be provided: 1) more than one treatment might be necessary for the complete destruction of *L. major* after 7 days from the first treatment and 2) phototherapy may need to be combined with other treatments (e.g., systemic therapies) (54). 

According to our measurements, the average depth of the lesion was almost 5 mm. It is widely believed that ICG-NIR therapy can serve as a valuable treatment option for CL since the reported penetration depth of NIR light is approximately 10 mm (55, 56). The synergistic effect of the ICG and NIR irradiation is expected to cause parasite destruction through apoptosis primarily (55).

This study was the first attempt to fight cutaneous leishmaniasis in Balb/c mice using ICG. One of the most significant challenges of PDT and PTT in animal models is establishing the optimal dosages of photosensitizers and light dose (fluence). Addressing this challenge will be crucial for translating findings into effective human treatments.

ICG concentration in both samples was 2 μM

Note: arrows indicate the position of both spectra at 808 nm.

ICG: Indocyanine green

ICG concentration was 2 μM

ICG: Indocyanine green

ICG concentration was 2 μM

ICG: Indocyanine green

Data are described as the mean ± SD (n = 3).

ICG: Indocyanine green

Data are denoted as mean ± SD value (n = 3).

**Table 1:** Combination index after irradiation with different fluences of NIR laser (power intensity= 2.5 W cm^-2^) and the same concentration of ICG solutions ± SD

**Fluence (J cm** ^-2^ **)**	**Combination index**	
	Free-ICG	ICG-loaded nanomicelles
**750**	1.32± 0.13	1.33±0.13
**1500**	2.62± 0.26	3.86± 0.38
**Mean CI ± SD**	1.97± 0.20	2.59± 0.25

Data are described as mean ± SD (n = 3). Notes: Groups statistically significant (*P*<0.05) in comparison with "control" (***) and "laser only" (#).

ICG: Indocyanine green

The fluence of the laser and ICG concentration were 1500 J cm^-2^ and 100 μg/ml, respectively (the data are presented as the mean± SEM). The number of mice in each group was 4.

ICG: Indocyanine green

The results are presented as the mean± SEM. The number of mice in each group was 4.

## Conclusion

Light-based technologies mediated by ICG may be a favorable choice for creating novel, affordable, and safe substances to fight *L. major*. This method shows great potential for future research. However, ICG tends to aggregate in aqueous solutions, which restricts its application in phototherapy. Therefore, a nanoparticulated micelle-based system was used to encapsulate ICG into biocompatible and biodegradable formulations, ultimately reducing the dimerization of ICG. This strategy led to a reduction in self-aggregation and, subsequently, a significant increase in the photophysical properties of ICG. *In vitro* and *in vivo *evaluations revealed a significant decrease in the cell survival rate and lesion size between the groups receiving ICG and laser irradiation simultaneously, compared with those in the other groups. PTT/PDT mediated by ICG-loaded nanomicelles is a promising and efficient non-invasive method for managing *Leishmania major*.

## Declaration

We have not used any AI tools or technologies to prepare this manuscript.
